# Pericardial Fat Radiomics to Predict Left Ventricular Involvement and Provide Incremental Prognostic Value in ARVC

**DOI:** 10.3390/diagnostics15243240

**Published:** 2025-12-18

**Authors:** Mengqi Guo, Jinyu Zheng, Weihui Xie, Binghua Chen, Dongaolei An, Ruoyang Shi, Jinyi Xiang, Lianming Wu

**Affiliations:** Department of Radiology, Renji Hospital, School of Medicine, Shanghai Jiao Tong University, Shanghai 200127, China; wy20190316@163.com (M.G.); zjinyu1108@163.com (J.Z.); xieweihui2020@163.com (W.X.); chenbinghua0311@163.com (B.C.); adalrenji@163.com (D.A.); ryshicn@shsmu.edu.cn (R.S.)

**Keywords:** arrhythmogenic right ventricular cardiomyopathy, heart, radiomics, magnetic resonance imaging

## Abstract

**Background/Objectives**: To explore the predictive value of pericardial fat tissue (PFT) radiomics for left ventricular (LV) involvement and major adverse cardiac events (MACE) in arrhythmogenic right ventricular cardiomyopathy (ARVC). **Methods:** In this retrospective multicenter study, LV involvement was assessed using cardiac magnetic resonance (CMR). A radiomic score (RS) derived from PFT was developed to predict LV involvement. The predictive accuracy of the RS was evaluated through receiver operating characteristic (ROC) analysis. Additionally, multivariable Cox regression analysis was employed to assess the prognosis across the entire dataset. Kaplan–Meier survival curves were used to evaluate the association between RS and MACE. **Results:** A total of 122 patients (mean age, 44 years ± 17; 76 male) were included, 90 for a development set and 32 for an external test set. The RS demonstrated good predictive performance for LV involvement in both the development and external test sets, with area under the curve (AUC) values of 0.771 and 0.785, respectively. Moreover, a high RS (≥−0.38) was independently associated with MACE during a median follow-up of 5 years (hazard ratio, 3.452; *p* < 0.001). Based on the right ventricular ejection fraction (RVEF) and RS, a simplified risk score was developed to categorize patients into three groups: high-risk (RVEF ≤ 40%, RS ≥ −0.38), intermediate-risk (RVEF ≤ 40%, RS < −0.38 or RVEF > 40%, RS ≥ −0.38), and low-risk (RVEF > 40%, RS < −0.38). **Conclusions:** The PFT radiomics can predict LV involvement and be associated with MACE in ARVC patients.

## 1. Introduction

Arrhythmogenic right ventricular cardiomyopathy (ARVC) is a complex genetic disorder characterized by progressive fibro-fatty replacement of the myocardium, predominantly affecting the right ventricle [1]. However, left ventricular (LV) involvement is increasingly being recognized, with significant implications for prognosis and management [2,3,4]. On cardiac magnetic resonance (CMR) imaging, late gadolinium enhancement (LGE) is a pivotal tool for assessing myocardial fibrosis and has been instrumental in evaluating LV involvement in ARVC, which is associated with a bad clinical outcome [5].

Recent advances in CMR technology have expanded the potential for comprehensive cardiac assessment, including the quantification and analysis of pericardial fat tissue (PFT) [2]. PFT, the visceral fat deposit surrounding the heart, has emerged as a novel biomarker in cardiovascular disease, influencing cardiac function and structure through mechanical, paracrine, and vasocrine effects. Studies have shown that the volume and characteristics of PFT are associated with various cardiac pathologies [6,7], suggesting a potential role in modulating cardiac morphology and function.

Given the established relationship between PFT and cardiac parameters, and the critical role of LV involvement for determining the prognosis of ARVC patients, this study aims to explore predictive value of PFT radiomics features for LV involvement in ARVC. Radiomics extracts numerous quantitative features from medical images and, when integrated with machine learning, offers a promising approach to assess the complex interactions between PFT and cardiac structures in a detailed and non-invasive manner [8]. By analyzing these features from routine cine CMR images, which are typically used to evaluate cardiac function, we hypothesize that PFT characteristics could serve as early indicators of LV involvement and potentially provide prognostic information beyond traditional imaging markers.

## 2. Materials and Methods

**Figure A1:**
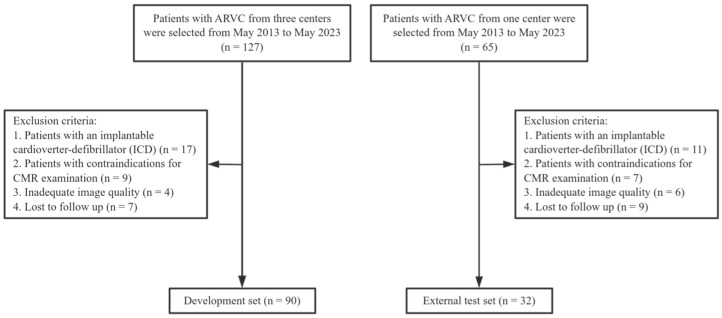
The flow chart of patient inclusion and exclusion.

This retrospective study enrolled 122 participants from four centers who met the 2010 Task Force Criteria (TFC) for ARVC [9] and underwent CMR imaging between May 2013 and May 2023. Patients were consecutively enrolled at each participating center based on the availability of complete CMR studies meeting quality criteria. Then, they were divided into a development set (*n* = 90, three centers) and an external test set (*n* = 32, one center). The 90 patients in the development set have been previously reported [10]. This prior article dealt with prognostic value of the right atrial strains in ARVC patients, whereas in this manuscript we report on the value of pericardial fat tissue radiomics. Patients with an implantable cardioverter-defibrillator (ICD) at baseline were excluded. Exclusion criteria were defined by contraindications to CMR that could compromise patient safety or image quality. Specifically, we excluded individuals with impaired renal function (glomerular filtration rate < 30 mL/min), claustrophobia, arrhythmia, or CMR-incompatible implants (Figure A1). Notably, a patient cohort defined solely by meeting the 2010 Task Force Criteria (TFC) for arrhythmogenic right ventricular cardiomyopathy (ARVC) almost certainly exhibits a non-uniform and skewed representation of the disease spectrum. This is an inherent and well-recognized limitation of these diagnostic criteria, which prioritizes specificity over sensitivity to avoid misdiagnosis. The 2010 TFC are heavily weighted toward identifying “manifest and often advanced disease”. They excel at detecting the “classic” RV-dominant form but systematically under-represent other presentations. On the one hand, major criteria (e.g., severe RV dysfunction/aneurysms, epsilon waves) typically only emerge after significant structural remodeling. Therefore, cohorts are enriched with patients in “Padua stages C/D” (symptomatic with arrhythmias or heart failure) while under-representing stages A/B (concealed/subclinical). On the other hand, the 2010 criteria lack major criteria for isolated LV abnormalities. Patients with predominant LV fibrosis, dysfunction, or inferolateral T-wave inversions may not score enough points for a “definite” diagnosis, leading to their exclusion. Gene-positive family members or individuals with minor abnormalities (e.g., isolated PVCs, localized minor wall motion issues) are also frequently excluded. And patients presenting with acute myocarditis-like symptoms (chest pain, troponin rise, LV involvement) without concurrent major RV criteria may be missed. More ARVC patients defined by updated 2020 Padua criteria are needed in future studies.

All scans were conducted on 3.0T scanners (Ingenia, Philips, Best, The Netherlands, and MAGNETOM Skyra, Siemens Healthcare, Erlangen, Germany). The CMR protocols are described in detail in Appendix A.1.

Two radiologists (XX with 13 years’ experience and XX with 10 years), who were blinded to clinical data, accessed all CMR images independently using commercially available software Cvi42 (version 5.11.3, Circle Cardiovascular Imaging Inc., Calgary, AB, Canada). For inconsistent results, further iterative discussions were conducted until a consensus was reached. To confirm ventricular function and volumes, right ventricular (RV) endocardial contours and LV epicardial and endocardial contours on short-axis cine images at end systole and end diastole were traced automatically by the software. To obtain RV end-systolic volume index (RVESVI) and RV end-diastolic volume index (RVEDVI), end-systolic volume and end-diastolic volume were adjusted according to body surface area (BSA). PFT area was measured from the horizontal long-axis cine images, and pericardial fat volume (PFV) was automatically calculated. RV LGE was achieved by full short-axis coverage of RV and evaluated qualitatively. LV LGE was determined by the line width technique. Wall motion abnormality (WMA) was accessed on 2/4-chamber and short-axis cine images. LV involvement was qualified when one or more of these following conditions were shown: LVEF < 50%, LV WMA, LGE with non-ischemic pattern, and LV fat infiltration, which was characterized by intramyocardial hyperintensity in SSFP images [2,11].

PFT was analyzed from end-diastolic cine images, with continuous short-axis slices segmented using the previously validated 3SUnet deep learning model [12]. Radiomics features were extracted using Pyradiomics [13], resulting in a total of 851 features. Given the importance of volume in the current PFT research [14], PFV was analyzed independently of the other radiomics features. All these radiomics features were computed from the original region of interest (ROI) and eight wavelet-transform filters. Details are described in Appendix A.2.

**Figure 1:**
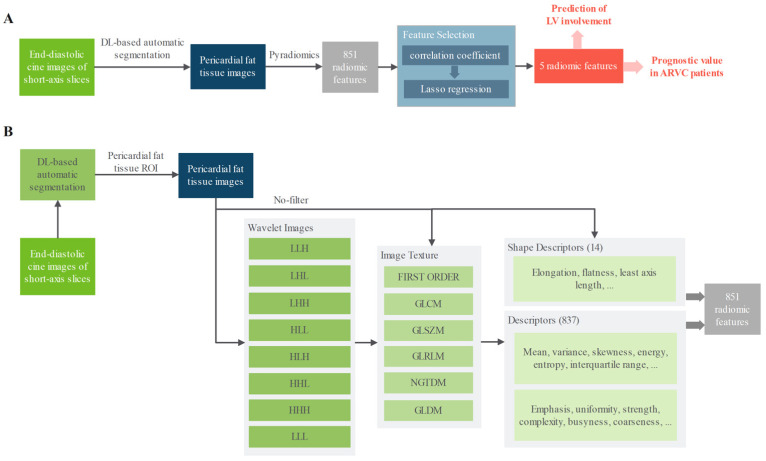
Block diagram of PFT radiomics feature extraction and feature selection. PFT—pericardial fat tissue. (**A**) Overall workflow; (**B**) detailed illustration of the PFT radiomics feature extraction step in (**A**).

For feature selection, we first removed parameters with collinearity greater than 0.8, reducing the dataset to 160 features. Least absolute shrinkage and selection operator (Lasso) regression was then applied. The hyper-parameter was determined through a fivefold cross validation. As a result, it yielded five features. The final radiomic score (RS) was derived from a logistic regression incorporating these five features to predict LV involvement on the development set (Figure 1).

All patients underwent a follow-up period with a median duration of 58.5 months. Major adverse cardiac events (MACE) were defined as appropriate ICD intervention, sudden cardiac death (SCD), and resuscitated cardiac arrest [15]. Clinical data were collected through periodic telephone contact or outpatient visits. In cases where ventricular fibrillation or ventricular tachycardia transpired above the pre-programmed threshold of the ICD (12 intervals at a heart rate exceeding 180 beats per minute), an appropriate ICD intervention was typically considered requisite. For patients with definite ARVC, the 5-year ARVC risk score was used to assess malignant ventricular arrhythmias risk, which was correlated with adverse cardiovascular events [15,16]. The ARVC risk score for each patient was computed using a set of parameters. These parameters encompassed age, male sex, cardiac syncope, the occurrence of non-sustained ventricular tachycardia (NSVT), the number of leads with inverted T waves (TWI), 24 h premature ventricular complex (PVC) count, and RVEF.

Forest plots were used to demonstrate the predictive value of variable characteristics for LV involvement. The predictive value of RS was assessed internally and externally by receiver operating characteristic (ROC) analysis. And the incremental predictive value of the RS beyond PFV was evaluated by the ROC curve. To determine the optimal cutoff value of RS for distinguishing high-risk (subsequently referred to as RS positive) from low-risk (subsequently referred to as RS negative) sets, the Youden index was utilized.

The prognostic value of the PFT radiomics features was evaluated in the data sets. The Youden index was used to determine the appropriate threshold of RS for distinguishing RS positive from RS negative patient sets. Multivariable and univariable Cox regression analyses were used to evaluate the RS, PFV, and conventional 5-year ARVC risk score for predicting MACE. Kaplan–Meier survival curves were applied to show the risk stratification value of the PFT radiomics features. The prognostic values of the RS and 5-year risk score were assessed with Harrell C index and compared by Net Reclassification Index (NRI). A mediation analysis was applied to explore the association between PFT and the occurrence of MACE. To assess the robustness of the RS against potential inter-scanner variability, a scanner-sensitivity analysis was performed. The distribution of the five constituent radiomic features and the final RS were compared between two scanner groups using the Independent-Samples T test. A statistically significant difference was defined if two-tailed *p* < 0.05. R software (version 4.4.0; www.R-project.org) and SPSS Statistics (version 27.0.1; IBM SPSS Inc., Chicago, IL, USA) were used to perform all the statistical analyses.

## 3. Results

### 3.1. Study Population Characteristics

This multicenter study enrolled 122 patients (mean age 44 ± 17 years; 76 males), divided into a development set and an external test set. Baseline characteristics for these sets are detailed in Table 1. In the development set, 63% of the participants were male, with an average age at diagnosis of 46 ± 16 years. In the external test set, 59% of the participants were male, with an average age at diagnosis of 40 ± 17 years. Significant differences were not found between the two sets regarding sex (*p* = 0.691), age (*p* = 0.110), PFV (*p* = 0.661), or other demographic characteristics. Most participants met at least one major diagnostic criterion of TFC. Over a median follow-up of 58.5 months, 42 of the 122 participants (34%) experienced MACE, with a higher incidence observed in the external test cohort (44% vs. 31%, *p* = 0.28). Demographic and clinical characteristics stratified by MACE are summarized in Table A1. Patients who developed MACE exhibited a higher prevalence of electrophysiological abnormalities, including recent cardiac syncope (21% vs. 6%, *p* = 0.012), 24 h PVC count (2533 vs. 990, *p* = 0.003), and NSVT (69% vs. 31%, *p* < 0.001). There were no significant differences in gender (60% vs. 64%, *p* = 0.65) or age (48 ± 16 vs. 43 ± 17, *p* = 0.11) between the groups with and without MACE. No statistically significant differences were observed in the RS across scanner types (*p* = 0.701), supporting its stability despite protocol heterogeneity.

### 3.2. CMR Findings

In the study population, RV systolic function was markedly impaired (RVEF, 31% ± 13%), while LVEF was relatively preserved (48% ± 15%). No significant differences were seen in most CMR parameters between the development and external test sets (Table 1). Patients who experienced MACE exhibited significantly reduced LVEF (36% ± 14% vs. 56% ± 8%, *p* < 0.001) and RVEF (23% ± 11% vs. 36% ± 13%, *p* < 0.001), along with a higher prevalence of LV WMA and LGE. RVEDVI and RVESVI were particularly greater in patients with MACE compared to those without (Table A1). Additionally, PFV was notably higher in the MACE group (*p* = 0.02).

### 3.3. Prediction of LV Involvement

**Figure 2:**
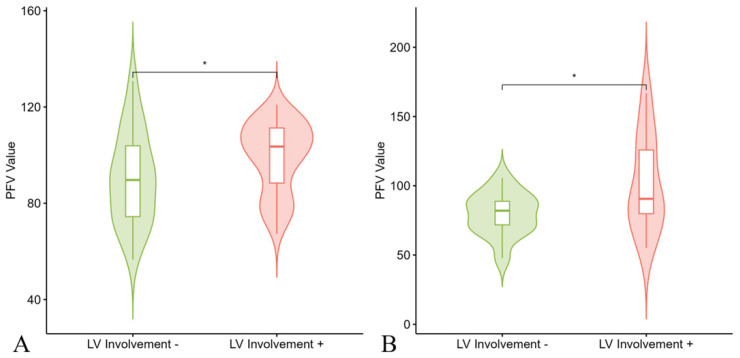
Violin plots for the distribution of the PFV value categorized by LV involvement. ARVC patients with LV involvement had higher PFV. (**A**) Development set; (**B**) external test set. PFV—pericardial fat volume, LV—left ventricular. An asterisk (*) marks a comparison where the difference is statistically significant, as determined by *t*-test.

**Figure 3:**
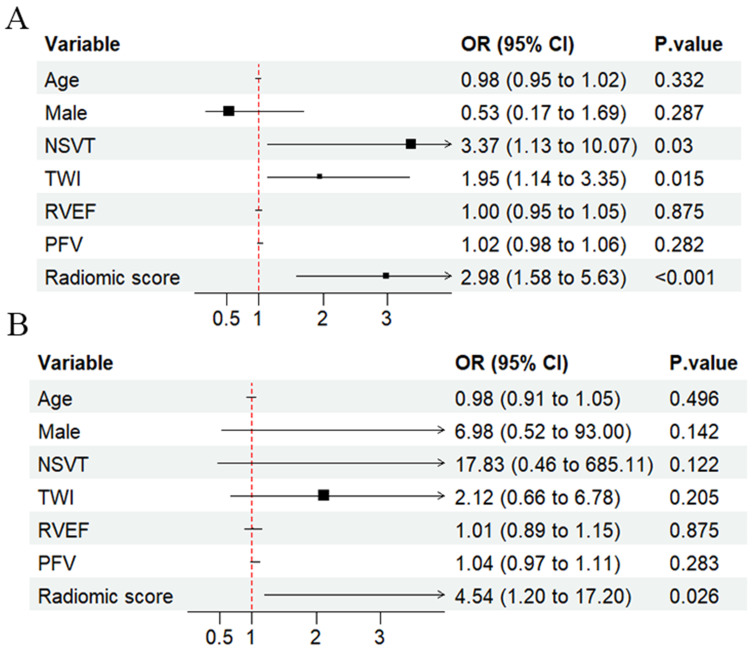
Forest plots of the prediction of LV involvement. RS remained an independent predictor for LV involvement in the multivariable logistic regression analysis. (**A**) Development set; (**B**) external test set. LV—left ventricular, RS—radiomic score. Black blocks (size indicates weight) and horizontal lines ("arrows") (95% CI) depict effect estimates. The red dashed line marks the null value (1.0).

**Figure 4:**
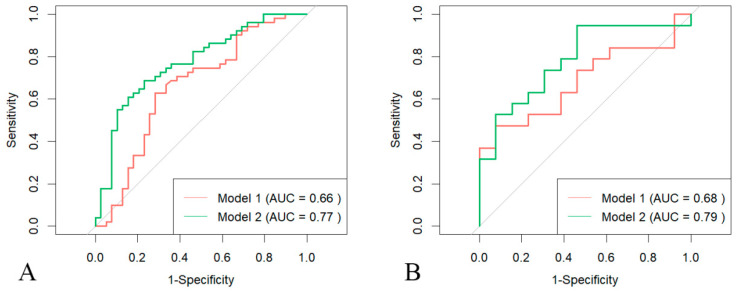
ROC curves of RS and conventional quantitative parameters (PFV) on CMR. RS (green) has higher discriminatory power to identify LV involvement compared with conventional quantitative metric (red). (**A**) Development set; (**B**) external test set. ROC—receiver operating characteristic curves, RS—radiomic score, PFV—pericardial fat volume, LV—left ventricular. The grey diagonal line represents the performance of a random classifier (no discriminative power).

**Figure A2:**
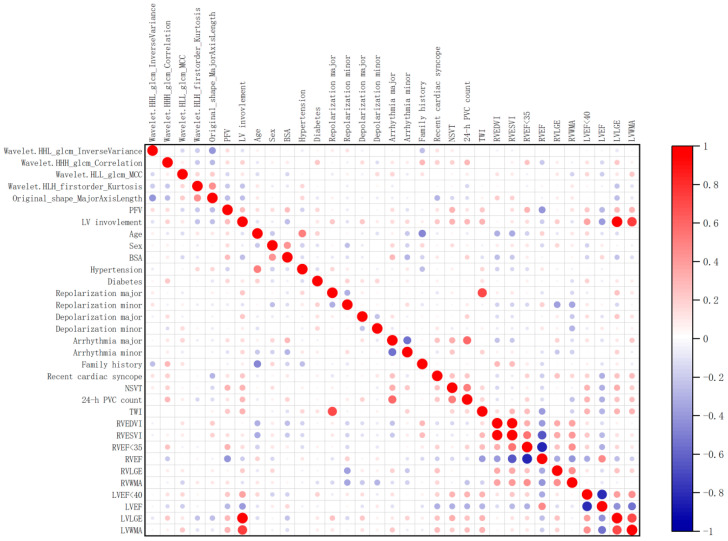
Correlogram showing the correlation among the selected radiomic features and patients’ clinical variables. The size of each circle is directly proportional to the absolute value of the correlation coefficient between the two variables it represents. Larger Circle = Stronger correlation (closer to +1 or −1). Smaller Circle = Weaker correlation (closer to 0). No Circle (or a tiny dot) = Very weak or no linear correlation.

**Figure A3:**
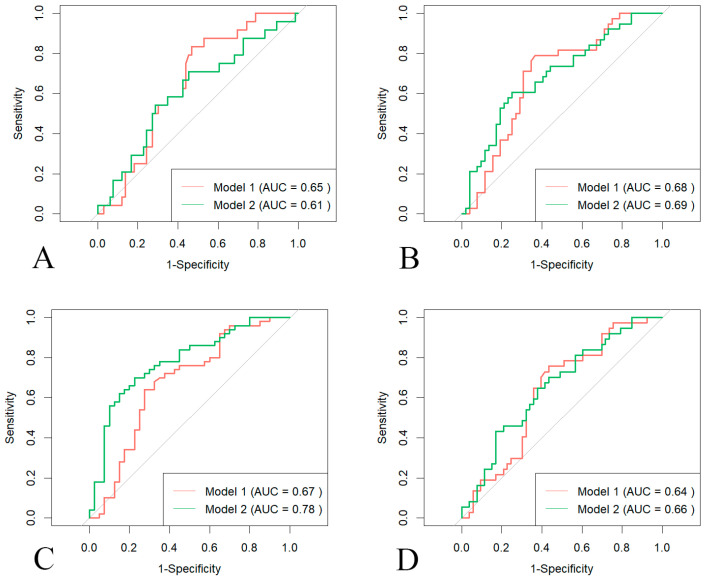
ROC curves of RS and conventional quantitative parameters (PFV) on CMR within development set. RS (green) has higher discriminatory power to identify non-ischemic LGE compared with conventional quantitative metric (red). (**A**) LVEF < 50%; (**B**) LV WMA; (**C**) non-ischemic LGE; (**D**) LV fat infiltration. ROC—receiver operating characteristic curves, RS—radiomic score, PFV—pericardial fat volume, LV—left ventricular. The grey diagonal line represents the performance of a random classifier (no discriminative power).

**Figure A4:**
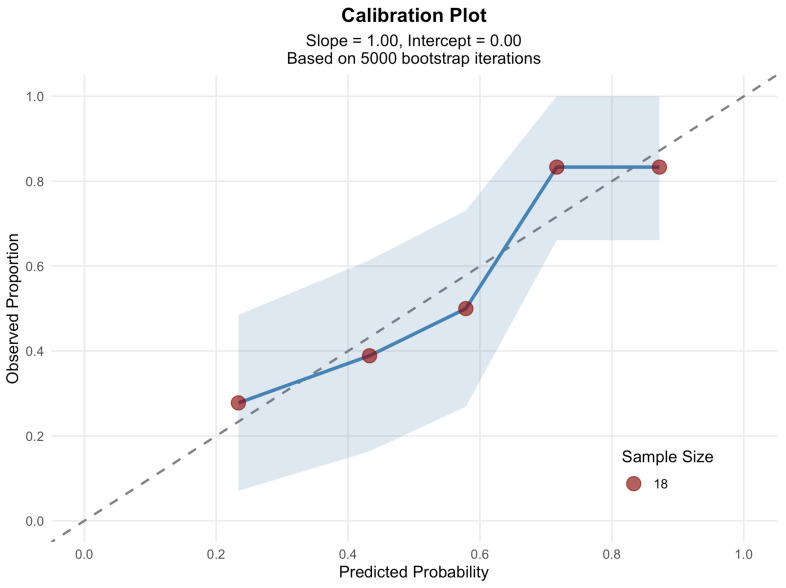
Calibration assessment of RS. The blue shaded region represents the 95% confidence interval for the estimated calibration curve, indicating the range within which the true calibration curve is likely to lie given the sample data. The dashed diagonal line represents the ideal of perfect calibration (predicted probability equals observed frequency).

To evaluate the predictive value of various characteristics for LV involvement, both multivariate and univariate logistic regression analyses were conducted. In the univariate analysis, electrophysiological features were significantly associated with LV involvement across both the development and external test cohorts, especially 24 h PVC count. Figure 2 illustrates that patients with LV involvement had higher PFV. Given this association, radiomics analysis was performed. The RS was much higher in the LV involvement group in both the development and test cohorts. Correlation analysis, depicted in Figure A2, shows that these selected features were correlated with electrophysiological features and both functional and anatomical abnormalities of the LV. In the multivariable logistic regression analysis (Figure 3), RS remained an independent predictor for LV involvement (OR, 4.75; 95% CI: 1.19, 18.91; *p* = 0.027). The predictive performance of the PFV model and RS model are presented in Figure 4. The RS model demonstrated a middling to good area under the curve (AUC) in the development (AUC, 0.771; 95% CI: 0.672, 0.870) and external test groups (AUC, 0.785; 95% CI: 0.622, 0.949) (Table 2 and Table A2). Figure A3 illustrates that the RS was most closely correlated with non-ischemic LGE among those with left ventricular damage. Bootstrap resampling (5000 iterations) was used to internally validate the model in our development set, with optimism-corrected metrics (AUC, 0.77, Brier score 0.20). The model was well-calibrated, as shown in a calibration plot (Figure A4) and supporting statistics.

### 3.4. Prognostic Value of Pericardial Fat Tissue

**Figure 5:**
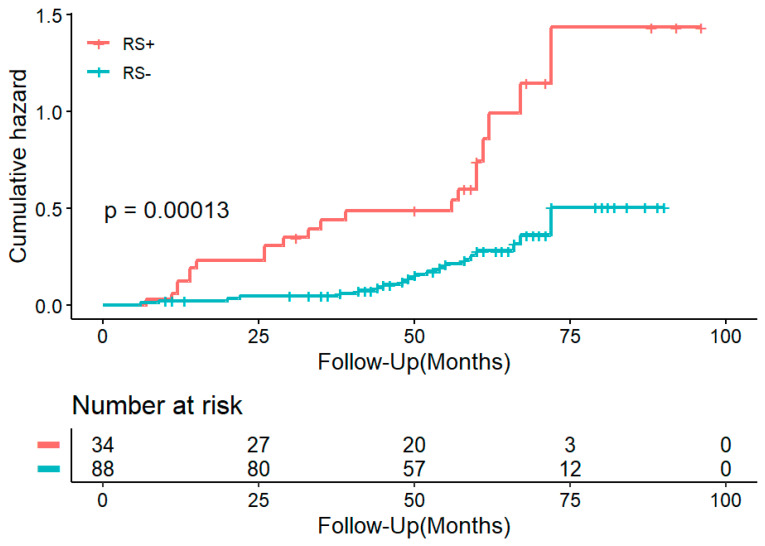
Kaplan–Meier curves show prognostic value of the RS. Curves for MACE stratified according to RS. RS+—RS positive; RS−—RS negative. RS—radiomic score, MACE—major adverse cardiac events.

**Figure 6:**
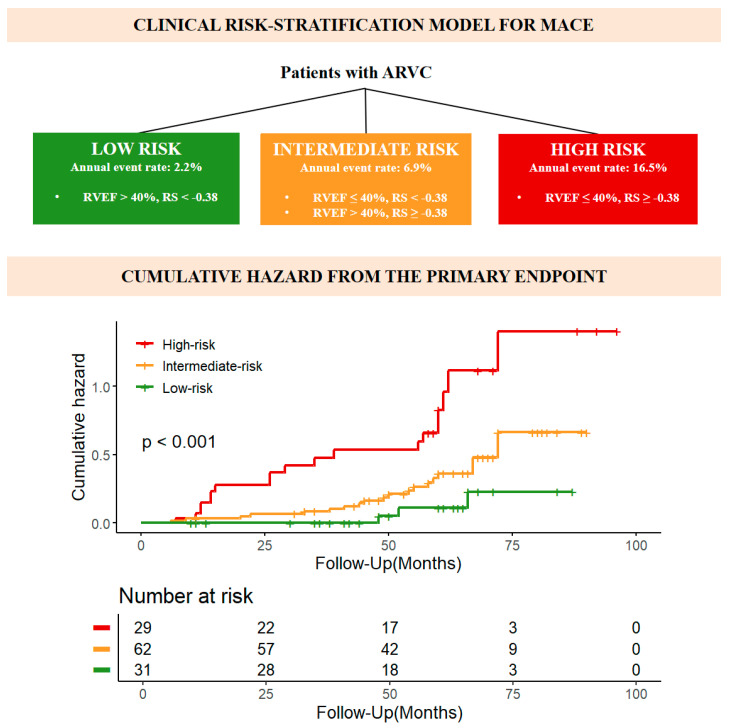
PFT radiomics for risk stratification in ARVC patients. PFT—pericardial fat tissue, ARVC—arrhythmogenic right ventricular cardiomyopathy.

The prognostic implication of PFT radiomics features was assessed across the entire dataset. Univariable Cox regression analysis revealed that individuals who are RS positive exhibited a twofold increased risk of MACE (hazard ratio [HR], 2.079; 95% CI: 1.495, 2.891; *p* < 0.001) compared to those who are RS negative. This association persisted even after adjusting for clinical variables (See Table 3). Kaplan–Meier survival curves indicated significant differences between groups that are RS positive and RS negative (Figure 5), allowing for stratification of patients based on RVEF and RS (Figure 6). A simplified risk score was developed, categorizing patients into three groups: high-risk (RVEF ≤ 40%, RS ≥ −0.38), intermediate-risk (RVEF ≤ 40%, RS < −0.38 or RVEF > 40%, RS ≥ −0.38), and low-risk (RVEF > 40%, RS < −0.38). The incremental prognostic value of the model combining the traditional 5-year risk score with RS (model 3) demonstrated improved goodness over the traditional 5-year risk score alone (model 1) (C index, 0.73 ± 0.08 vs. 0.70 ± 0.08; *p* < 0.001) (Table 4). Furthermore, the prognostic value of adding RS to RVEF was superior to that of adding LV involvement to RVEF (C index, 0.76 ± 0.07 vs. 0.73 ± 0.07; *p* < 0.001) (Table A3). Mediation analysis showed that LVEF and RVEF accounted for 41.5% and 33.3%, respectively, of the total variance explained in the observed associations, with detailed decomposition provided in Table A4. These results are consistent with a hypothesized functional relationship involving PFT.

### 3.5. Interobserver and Intraobserver Variability of Radiomic Features

The interobserver reproducibility was good to excellent for the selected radiomics features, with Original-Shape-Major Axis Length being the most reproducible feature (ICC, 0.77; Table A5). For Observer 1, all the selected features showed good to excellent intraobserver reproducibility. Wavelet.HLL-GLCM-MCC was the most reproducible feature (ICC, 0.82; Table A6). All these features also had excellent intraobserver reproducibility for Observer 2, with Wavelet.HHL-GLCM-InverseVariance being the most reproducible feature (ICC, 0.81; Table A6). Nearly all the selected radiomics features had good to excellent reproducibility.

## 4. Discussion

In this study, we explored the predictive value of PFT for LV involvement in a large cohort of patients with definite ARVC and explored its prognostic value. The principal results of our research are presented as follows: (a) The RS was able to predict LV involvement with a middling to good AUC both on the development and external test sets; (b) Individuals who are RS positive exhibited a twofold increased risk of MACE compared to those who are RS negative; (c) RS demonstrated incremental prognostic value above the 5-year risk score; (d) A simplified risk score was developed categorizing patients into three groups based on RVEF and RS. This study underlies the potential of the PFT in predicting LV involvement and providing prognostic value.

LV involvement has been increasingly recognized in ARVC, carrying significant implications for prognosis and management [1,17]. A study has reviewed the current literature and compare these cohorts of patients, confirming left ventricular functional change in arrhythmogenic right ventricular cardiomyopathy [18]. Consequently, assessing LV involvement is critical in the ARVC population. However, typical evaluation of LV involvement is time-consuming, labor-intensive, and is prone to subjective interpretation, particularly as it requires the use of LGE to assess myocardial fibrosis.

LV involvement is crucial for the prognosis of ARVC, which suggests that some factors related with LV involvement may also provide prognostic value in ARVC patients. A multicenter retrospective study and a genetic analysis study including 27 probands have shown that some plasma biomarkers and specific genetic mutations may predict LV involvement of ARVC patients [19,20]. In addition, atrial strain and depolarization voltage mapping were also regarded as LV involvement biomarkers in some research. A study consisting of 209 patients with pulmonary hypertension demonstrated that LA reservoir function showed good diagnostic performance to identify patients with left cardiac involvement evident at rest (AUC 0.81) [21]. However, the acquisition of these factors is invasive or complex, which is difficult to apply in clinical practice. Instead of that, the RS in our study is a non-invasive biomarker and more accessible.

PFT has been proven to be associated with cardiac structure and function and can be routinely assessed through cine images [22]; our study explored the potential of PFT radiomics features to predict LV involvement in ARVC patients. The RS demonstrated a good predictability in the development set and external test set. Of note, we utilized a fully automated and externally validated method [12] to extract PFT from widely available cine sequence, facilitating the analytic pipeline. Therefore, our study is also a meaningful attempt to apply this DL-based automatic segmentation method for clinical research.

A retrospective study including 254 adults and a Multi-Ethnic Study of Atherosclerosis including 3032 participants have shown that PFV is associated with dysfunction of the atrium and right ventricle, even in healthy populations [23,24]. Importantly, increased PFT may directly lead to diastolic dysfunction. And the structural and functional changes in the heart may play a potential mechanic role for PFT in early heart failure. These findings suggest a possible prognostic role of PFT in ARVC patients. In our study, the RS provided incremental prognostic value over the well-known 5-year risk score model. The risk stratification utility of RS was described in our study, and mediation analysis revealed a possible functional and anatomical pathway between PFT and adverse outcomes.

PFT exhibits high metabolic activity and has been associated with elevated local production of pro-inflammatory mediators, including tumor necrosis factor-α, interleukin-1β, and interleukin-6 [25]. These inflammatory mediators contribute to the development of cardiac conditions such as pericarditis and myocarditis, thereby accelerating the occurrence of MACE. In addition, increased PFT is linked to various structural alterations in the heart, which may enhance the risk of ventricular arrhythmia (VA) in ARVC patients. Notably, as PFT expands, there is evidence of fatty infiltration in the ventricular myocardium and atrial septum, which could lead to electromechanical dysfunction [26,27]. The volume and characteristics of PFT may lead to myocardial steatosis and extracellular infiltration of fat between myocardial fibers, resulting in cardiac dysfunction. Although the distinct role of PFT in the development of ARVC is not well elucidated, PFT is correlated with LV involvement and is prospectively associated with future MACE in ARVC patients.

The current clinical challenge in the care of ARVC patients lies in identifying which individuals would benefit most from lifestyle modifications, implantable cardioverter-defibrillators, and antiarrhythmic medication [28]. According to the 2019 HRS experts consensus [11], prevention of SCD is possible with ICDs, and identifying patients at risk of SCD is essential to target those who should receive these devices. Programmed ventricular stimulation (PVS), LV involvement, and individual genes significantly improved risk stratification in ARVC patients compared to clinical risk calculators [2,15,29]. An electrophysiology study suggested that the presence and extent of low QRS voltage may serve as a prognostic marker for heart failure-related death or heart transplantation in patients with ARVC. Our research further stratified the risk level of ARVC based on RVEF and the characteristics of PFT on CMR. Compared with previous studies, our stratification criteria, RVEF and CMR cine imaging, are commonly used for evaluating ARVC patients and are easier to obtain, thereby simplifying the evaluation process and facilitating broader application.

Although some positive results have been achieved, there are certain limitations that should be discussed. First, bias of selection could not be ignored for its retrospective design. And arrhythmia-induced motion artifacts could theoretically impact the precision of myocardial segmentation and the derived radiomic features because of the absence of CMR frame-quality control for arrhythmia frames. Our study included data from multiple 3.0T scanners with variations in acquisition parameters. While the scanner-sensitivity analysis did not detect a significant batch effect on the final RS in our cohort, we acknowledge that undetected or non-linear batch effects may still exist and represent a potential limitation. Feature harmonization techniques (e.g., ComBat) were considered; however, given the moderate cohort size and the risk of over-correction, we prioritized demonstrating the intrinsic robustness of the selected features. Future multicenter validations should implement prospective imaging protocol standardization or advanced harmonization methods to further mitigate this concern. Second, the sample size was small in the external test set, limiting its statistical power. The overall small population size may not be accurately reflective of PFT and other characteristics in ARVC patients. Further prospective and large-scale validations are warranted to confirm these findings in multi-ethnic cohorts. Third, our study lacked relevant genetic findings. Ethical and regulatory issues make it difficult to access. Also, most ARVC patients are gene-elusive, making gene testing less cost-effective. While access to comprehensive genetic data remains a practical challenge, future validation of our model must prioritize the inclusion of genotyping. This is critical to establish whether the RS provides robust prognostic value independent of the specific underlying causative mutation. Finally, the biologic meaning of radiomics features is not fully understood. Based on the established pathophysiological link between dyslipidemia and atrial fibrillation [30], our findings extend this conceptual framework to ARVC. As illustrated in Figure A2, the five radiomic features we selected correlate significantly with electrophysiological parameters, as well as with functional and anatomical abnormalities of the left ventricle. This supports the rationale that these imaging-derived features capture relevant histopathological properties of PFT. We propose that, through mechanisms involving systemic inflammation, oxidative stress, and autonomic imbalance, these features modulate the extent of myocardial involvement and, consequently, influence clinical outcomes in patients with ARVC. Given this situation, future studies should pay more attention to the gap between the histological characteristics and radiomics of PFT. It is important to note that radiomic features are mathematical descriptors of image texture and patterns. Their direct biological correlates, while plausibly linked to inflammation, fibrosis, or fat infiltration as discussed, remain inferential. We should also avoid over-interpreting specific texture patterns as directly reflecting discrete biological processes without histological validation. While the RS demonstrated modest but significant incremental prognostic value in our cohort, its translation to clinical practice mandates validation in larger, prospective, multicenter studies with standardized imaging protocols.

## 5. Conclusions

In summary, this study shows a radiomic analysis of PFT obtained from CMR, which demonstrated an independent correlation with LV involvement in ARVC patients. The RS offers supplementary predictive value for adverse cardiovascular outcomes. It shows promise as a non-invasive imaging biomarker for risk stratification in clinical settings. Nevertheless, the current evidence base is limited, and large-scale prospective studies are required to confirm these findings. Moreover, future research endeavors should focus on examining the dynamic changes in radiomics features over time. Uncovering how these features evolve can provide deeper insights into disease progression. Future studies should also investigate the dynamics of pericardial tissue remodeling by employing 4D (time-resolved) radiomic analysis or by correlating radiomic features from multiparametric imaging sequences (e.g., T1 mapping, extracellular volume fraction) to capture signals of active disease progression, rather than relying on static anatomic assessments alone. Additionally, exploring their potential in guiding treatment decisions could be a valuable avenue of study, facilitating more personalized and effective therapeutic strategies for patients.

## Figures and Tables

**Table 1:** Study population characteristics.

Characteristic	Development Set (*n* = 90 Patients)	External Test Set (*n* = 32 Patients)	*p* Value
Clinical characteristics			
Age (y)	46 ± 16	40 ± 17	0.110
Male	57/90 (63)	19/32 (59)	0.691
BSA	1.65 ± 0.15	1.75 ± 0.18	0.010
Hypertension	21/90 (23)	3/32 (9)	0.088
Diabetes	5/90 (6)	1/32 (3)	0.585
Clinical presentation			
Recent cardiac syncope	10/90 (11)	4/32 (13)	0.832
NSVT	41/90 (46)	13/32 (41)	0.630
24 h PVC count	1503 (390–3061)	2453 (936–3793)	0.037
Leads with anterior and inferior TWI	2 (1–3)	2 (1–3)	0.881
5 yr ARVC risk score	0.23 (0.12–0.41)	0.24 (0.13–0.45)	0.710
Clinical phenotype			
Repolarization criteria			
Minor	23/90 (26)	9/32 (28)	0.777
Major	19/90 (21)	7/32 (22)	0.928
Depolarization criteria			
Minor	39/90 (43)	11/32 (34)	0.376
Major	6/90 (7)	5/32 (16)	0.129
Arrhythmia criteria			
Minor	39/90 (43)	17/32 (53)	0.340
Major	25/90 (28)	4/32 (13)	0.081
Structural criteria			
Minor	35/90 (39)	8/32 (25)	0.158
Major	74/90 (82)	22/32 (69)	0.110
Family history	22/90 (24)	7/32 (22)	0.769
CMR parameters			
LVEF	48.17 ± 14.63	52.03 ± 12.31	0.185
LV LGE presence	50/90 (56)	16/32 (50)	0.588
LV WMA	38/90 (42)	7/32 (22)	0.040
RVEF	31.13 ± 12.83	33.95 ± 17.55	0.410
RV LGE presence	59/90 (66)	17/32 (53)	0.213
RV WMA	72/90 (80)	23/32 (72)	0.342
RVEDVI (mL/m^2^)	119.86 ± 44.95	115.73 ± 61.32	0.687
RVESVI (mL/m^2^)	85.05 ± 41.29	81.70 ± 56.14	0.721
PFV	95.31 ± 17.12	92.84 ± 29.96	0.661

Values are median (IQR) or *n* (%). Continuous variables were compared. BSA—body surface area, NSVT—non-sustained ventricular tachycardia, PVC—premature ventricular complex, TWI—inverted T-wave, ARVC—arrhythmogenic right ventricular cardiomyopathy, CMR—cardiac magnetic resonance, LVEF—left ventricular ejection fraction, LV—left ventricle, LGE—late gadolinium enhancement, WMA—wall motion abnormality, RVEF—right ventricular ejection fraction, RV—right ventricle, RVEDVI—right ventricular end-diastolic volume index, RVESVI—right ventricular end-systolic volume index, PFV—pericardial fat volume.

**Table 2:** Diagnostic performance of variable characteristics for prediction of LV Involvement in the development and external test sets.

	Development Set (*n* = 90 Patients)		External Test Set (*n* = 32 Patients)	
Variable	AUC	95% CI	AUC	95% CI
Recent cardiac syncope	0.575	0.517–0.634	0.605	0.511–0.699
NSVT	0.653	0.554–0.752	0.713	0.560–0.866
24 h PVC count	0.718	0.605–0.831	0.623	0.418–0.829
Leads with anterior and inferior TWI	0.677	0.568–0.787	0.692	0.510–0.874
RVEF	0.605	0.480–0.730	0.648	0.449–0.846
RV LGE presence	0.603	0.504–0.703	0.753	0.596–0.910
RV WMA	0.459	0.377–0.541	0.457	0.297–0.618
RVEDVI (mL/m^2^)	0.546	0.425–0.666	0.688	0.496–0.880
RVESVI (mL/m^2^)	0.583	0.462–0.704	0.773	0.595–0.951
PFV	0.658	0.539–0.777	0.684	0.497–0.871
RS	0.771	0.672–0.870	0.785	0.622–0.949

LV—left ventricular, NSVT—non-sustained ventricular tachycardia, PVC—premature ventricular complex, TWI—inverted T-wave, RVEF—right ventricular ejection fraction, RV—right ventricle, LGE—late gadolinium enhancement, WMA—wall motion abnormality, RVEDVI—right ventricular end-diastolic volume index, RVESVI—right ventricular end-systolic volume index, PFV—pericardial fat volume, RS—radiomic score.

**Table 3:** Univariable and multivariable Cox regression analyses to predict subsequent MACE in the study cohort.

	Univariate Analyses		Multivariate Analysis	
Variable	HR	*p* Value	HR *	*p* Value
Clinical characteristics				
Age (y)	1.009 (0.991–1.028)	0.338	…	…
Male	0.805 (0.434–1.494)	0.492	…	…
BSA	0.225 (0.030–1.664)	0.144	…	…
Hypertension	0.546 (0.230–1.300)	0.172	…	…
Diabetes	1.479 (0.352–6.225)	0.593	…	…
Clinical presentation				
Recent cardiac syncope	2.308 (1.103–4.827)	0.026	3.091 (1.412–6.766)	0.005
NSVT	3.713 (1.920–7.180)	<0.001	4.027 (2.027–7.999)	<0.001
24 h PVC count	1.000 (1.000–1.000)	0.001	1.000 (1.000–1.000)	<0.001
Leads with anterior and inferior TWI	1.424 (1.179–1.721)	<0.001	1.692 (1.347–2.124)	<0.001
5 yr ARVC risk score	13.431 (4.224–42.706)	<0.001	64.847 (15.402–273.023)	<0.001
CMR parameters				
LVEF	0.942 (0.925–0.959)	<0.001	0.942 (0.924–0.961)	<0.001
LV LGE presence	3.409 (1.628–7.138)	0.001	3.499 (1.632–7.500)	0.001
LV WMA	2.353 (1.265–4.379)	0.007	2.245 (1.179–4.276)	0.014
RVEF	0.942 (0.917–0.968)	<0.001	0.938 (0.914–0.964)	<0.001
RV LGE presence	1.540 (0.774–3.066)	0.219	1.507 (0.748–3.038)	0.251
RV WMA	1.310 (0.606–2.832)	0.493	1.147 (0.507–2.598)	0.742
RVEDVI (mL/m^2^)	1.012 (1.006–1.019)	<0.001	1.012 (1.005–1.019)	0.001
RVESVI (mL/m^2^)	1.014 (1.007–1.021)	<0.001	1.016 (1.008–1.024)	<0.001
PFV	1.017 (1.002–1.032)	0.024	1.017 (1.002–1.033)	0.027
RS	3.452 (1.778–6.703)	<0.001	3.723 (1.872–7.401)	<0.001

MACE—major adverse cardiac events, BSA—body surface area, NSVT—non-sustained ventricular tachycardia, PVC—premature ventricular complex, TWI—inverted T-wave, ARVC—arrhythmogenic right ventricular cardiomyopathy, CMR—cardiac magnetic resonance, LVEF—left ventricular ejection fraction, LV—left ventricle, LGE—late gadolinium enhancement, WMA—wall motion abnormality, RVEF—right ventricular ejection fraction, RV—right ventricle, RVEDVI—right ventricular end-diastolic volume index, RVESVI—right ventricular end-systolic volume index, PFV—pericardial fat volume, RS—radiomic score. * HR was adjusted for clinical variables, including age, sex, body surface area, hypertension, and diabetes.

**Table 4:** Incremental prognostic value of the RS.

Prediction Model	C Index	Net Reclassification Index	*p* Value
Model 1: 5-year ARVC risk score	0.70 ± 0.08	NA	
Model 2: RS	0.67 ± 0.10	NA	
Model 3: model 1 + model 2	0.73 ± 0.08	Model 1 vs. Model 3	
		0.079 (0.018–0.412)	<0.001
		Model 2 vs. Model 3	
		0.315 (0.033–0.468)	<0.001

RS—radiomic score, ARVC—arrhythmogenic right ventricular cardiomyopathy.

**Figure A5:**
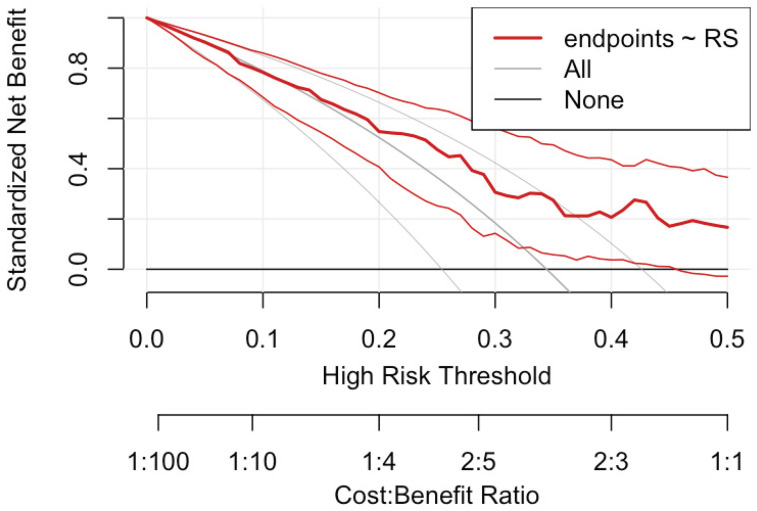
The Decision Curve Analysis of RS.

## Data Availability

The data presented in this study are available on request from the corresponding author. The data are not publicly available due to local policies.

## Abbreviations

The following abbreviations are used in this manuscript:

PFT	pericardial fat tissue
LV	left ventricular
ARVC	arrhythmogenic right ventricular cardiomyopathy
MACE	major adverse cardiac events
CMR	cardiac magnetic resonance
RS	radiomic score
ROC	receiver operating characteristic
PFV	pericardial fat volume
AUC	area under the curve
RVEF	right ventricular ejection fraction

## Appendix A

### Appendix A.1. CMR Acquisition

All examinations were performed on 3.0T scanners (Ingenia, Philips, Best, The Netherlands and MAGNETOM Skyra, Siemens Healthcare, Erlangen, Germany) using a 12-element phased-array coil. The CMR protocol consisted on cine imaging with complete RV coverage in short, 2-, 3-, 4-chamber views of 30 frames using a balanced steady-state free precession, T2-weighted short-tau triple inversion recovery (T2WI-STIR) sequence and LGE using phase-sensitive inversion recovery, which was acquired 10 min after administering gadolinium with Gd-DTPA (0.15 mmol/kg, diethylenetriaminepentacetate; Bayer Schering Pharma AG, Berlin, Germany or 0.3 mmol/kg, Adobenate Dimeglumine; Bayer Schering Pharma AG, Berlin, Germany).

Ingenia, Philips, Best, The Netherlands

Cine parameters were as follows: field of view (FOV), 300 × 300 mm^2^; voxel size, 2 × 1.75 × 6 mm^3^; flip angle (FA), 45°; bandwidth, 1410 Hz/pixel; repetition time (TR), 3.2 ms; echo time (TE), 1.6 ms.

T2WI-STIR parameters were as follows: FOV, 300 × 300 mm^2^; voxel size, 1.55 × 1.9 × 10 mm^3^; flip angle, 90°; bandwidth, 980 Hz/pixel; TR/TE, 2R-R/90 ms.

LGE parameters were as follows: FOV, 300 × 300 mm^2^; voxel size, 1.8 × 1.9 × 10 mm^3^; flip angle, 25°; bandwidth, 830 Hz/pixel; TR/TE, 4/2 ms.

MAGNETOM Skyra, Siemens Healthcare, Erlangen, Germany

Cine parameters were as follows: FOV, 360 × 360 mm^2^; voxel size, 1.7 × 1.7 × 8 mm^3^; flip angle, 46°; bandwidth, 965 Hz/pixel; TR/TE, 3.2/1.39 ms.

T2WI-STIR parameters were as follows: FOV, 360 × 360 mm^2^; voxel size, 1.4 × 1.4 × 8 mm^3^; flip angle, 160°; bandwidth, 930 Hz/pixel; TR/TE, 750/111 ms.

LGE parameters were as follows: FOV, 360 × 360 mm^2^; voxel size, 1.8 × 1.8 × 8 mm^3^; flip angle, 12°; bandwidth, 789 Hz/pixel; TR/TE, 3.2/1.27 ms.

### Appendix A.2. Pericardial Fat Tissue Analysis

Additional radiomic parameters included 18 first-order statistics, 14 shape features, and 75 texture features including 24 gray-level co-occurrence matrix (GLCM), 16 gray-level size zone matrix (GLSZM), 16 gray-level run-length matrix (GLRLM), 5 neighboring gray tone difference matrix (NGTDM), and 14 gray-level dependence matrix (GLDM).

**Table A1:** Study population characteristics according to MACE.

Characteristic	All Patients (*n* = 122)	Patients with MACE (*n* = 42)	Patients Without MACE (*n* = 80)	*p* Value
Clinical characteristics				
Age (y)	44 ± 17	48 ± 16	43 ± 17	0.114
Male	76/122 (62)	25/42 (60)	51/80 (64)	0.647
BSA	1.68 ± 0.16	1.64 ± 0.15	1.70 ± 0.16	0.044
Hypertension	24/122 (20)	6/42 (14)	18/80 (23)	0.278
Diabetes	6/122 (5)	2/42 (5)	4/80 (5)	0.954
Clinical presentation				
Recent cardiac syncope	14/122 (12)	9/42 (21)	5/80 (6)	0.012
NSVT	54/122 (44)	29/42 (69)	25/80 (31)	<0.001
24 h PVC count	1803 (528–3406)	2533 (1694–3846)	990 (381–2934)	0.003
Leads with anterior and inferior TWI	2 (1–3)	3 (2–3)	1 (1–2)	<0.001
5 yr ARVC risk score	0.24 (0.12–0.42)	0.37 (0.23–0.58)	0.19 (0.09–0.32)	<0.001
Clinical phenotype				
Repolarization criteria				
Minor	32/122 (26)	12/42 (29)	20/80 (25)	0.670
Major	26/122 (21)	8/42 (19)	18/80 (23)	0.658
Depolarization criteria				
Minor	50/122 (41)	17/42 (41)	33/80 (41)	0.934
Major	11/122 (9)	6/42 (14)	5/80 (6)	0.141
Arrhythmia criteria				
Minor	56/122 (46)	22/42 (52)	34/80 (43)	0.298
Major	29/122 (24)	9/42 (21)	20/80 (25)	0.660
Structural criteria				
Minor	43/122 (35)	12/42 (29)	31/80 (39)	0.264
Major	96/122 (79)	33/42 (79)	63/80 (79)	0.982
Family history	29/122 (24)	14/42 (33)	15/80 (19)	0.072
CMR Parameters				
LVEF	49.18 ± 14.11	36.27 ± 14.06	55.96 ± 8.21	<0.001
LV LGE presence	66/122 (54)	33/42 (79)	33/80 (41)	<0.001
LV WMA	45/122 (37)	25/42 (60)	20/80 (25)	<0.001
RVEF	31.87 ± 14.20	23.10 ± 11.16	36.47 ± 13.48	<0.001
RV LGE presence	76/122 (62)	31/42 (74)	45/80 (56)	0.057
RV WMA	95/122 (78)	34/42 (81)	61/80 (76)	0.552
RVEDVI (mL/m^2^)	118.78 ± 49.53	136.99 ± 67.64	109.22 ± 33.37	0.015
RVESVI (mL/m^2^)	84.17 ± 45.43	105.23 ± 59.04	73.12 ± 31.51	0.002
PFV	94.66 ± 21.13	100.61 ± 24.62	91.54 ± 18.67	0.024

Values are median (IQR) or *n* (%). Continuous variables were compared. MACE—major adverse cardiac events, BSA—body surface area, NSVT—non-sustained ventricular tachycardia, PVC—premature ventricular complex, TWI—inverted T-wave, ARVC—arrhythmogenic right ventricular cardiomyopathy, CMR—cardiac magnetic resonance, LVEF—left ventricular ejection fraction, LV—left ventricle, LGE—late gadolinium enhancement, WMA—wall motion abnormality, RVEF—right ventricular ejection fraction, RV—right ventricle, RVEDVI—right ventricular end-diastolic volume index, RVESVI—right ventricular end-systolic volume index, PFV—pericardial fat volume.

**Table A2:** The predictive ability of all models for LV involvement.

	AUC (95% CI)	*p* Value	Accuracy (%; [95% CI])	Sensitivity (%; [95% CI])	Specificity (%; [95% CI])	F1 Score
Development Set						
PFV model	0.658 [0.539–0.777]	…	66.67 (60/90; [57.78–76.67])	74.42 [62.35–88.37]	59.57 [45.82–73.32]	0.681
RS model	0.771 [0.672–0.870]	0.051	72.22 (63/90; [63.33–81.11])	79.55 [67.98–91.74]	65.22 [51.86–80.43]	0.737
External Test Set						
PFV model	0.684 [0.497–0.871]	…	65.63 (21/32; [50.00–84.38])	90.00 [80.00–113.33]	54.55 [34.09–75.76]	0.621
RS model	0.785 [0.622–0.949]	0.193	78.13 (25/32; [65.63–93.75])	75.00 [58.33–92.84]	87.50 [75.00–115.00]	0.837

PFV—pericardial fat volume, LV—left ventricular, RS—radiomic score.

**Table A3:** Comparison of RS and LV involvement in prognosis.

Prediction Model	C Index	Model 1 vs. Model 2	
		Net Reclassification Index	*p* Value
Model 1: LV involvement + RVEF	0.73 ± 0.07	0.136 (0.002–0.306)	<0.001
Model 2: RS + RVEF	0.76 ± 0.07		

RS—radiomic score, LV—left ventricular, RVEF—right ventricular ejection fraction.

**Table A4:** Mediating effects of RS on MACE with the mediators LVEF and RVEF.

	Direct Effect	*p* Value	Indirect Effect	*p* Value	Mediated Proportion	*p* Value
LVEF	0.186 (0.055, 0.310)	0.002	0.133 (0.032, 0.250)	0.008	0.415 (0.136, 0.740)	0.008
RVEF	0.212 (0.049, 0.380)	0.012	0.108 (0.039, 0.190)	<0.001	0.333 (0.119, 0.720)	0.002

RS—radiomic score, MACE—major adverse cardiac events.

**Table A5:** Interobserver reproducibility of selected PFT radiomic features for predicting LV involvement.

PFT Radiomic Features	ICC	95% CI
Wavelet.HHL-GLCM-InverseVariance	0.72	[0.56–0.81]
Wavelet.HHH-GLCM-Correlation	0.69	[0.54–0.80]
Wavelet.HLL-GLCM-MCC	0.74	[0.58–0.83]
Wavelet.HLH-First Order-Kurtosis	0.64	[0.49–0.78]
Original-Shape-Major Axis Length	0.77	[0.55–0.84]

PFT—pericardial fat tissue, LV—left ventricular, GLCM—gray-level co-occurrence matrix.

**Table A6:** Intraobserver reproducibility of selected PFT radiomic features for predicting LV involvement.

	Observer 1		Observer 2	
PFT Radiomic Features	ICC	95% CI	ICC	95% CI
Wavelet.HHL-GLCM-InverseVariance	0.67	[0.52–0.80]	0.81	[0.68–0.87]
Wavelet.HHH-GLCM-Correlation	0.72	[0.53–0.81]	0.73	[0.53–0.84]
Wavelet.HLL-GLCM-MCC	0.82	[0.61–0.91]	0.80	[0.69–0.88]
Wavelet.HLH-First Order-Kurtosis	0.69	[0.50–0.79]	0.77	[0.63–0.90]
Original-Shape-Major Axis Length	0.64	[0.50–0.81]	0.74	[0.57–0.86]

PFT—pericardial fat tissue, GLCM—gray-level co-occurrence matrix.

**Table A7:** The pairwise Spearman correlations among the five final radiomic features.

Feature 1	Feature 2	Correlation	Interpretation
wavelet.HHL_glcm_InverseVariance	original_shape_MajorAxisLength	−0.484	Moderate
wavelet.HLH_firstorder_Kurtosis	original_shape_MajorAxisLength	0.363	Moderate
wavelet.HHH_glcm_Correlation	original_shape_MajorAxisLength	−0.32	Moderate
wavelet.HHH_glcm_Correlation	wavelet.HLH_firstorder_Kurtosis	−0.266	Weak
wavelet.HHL_glcm_InverseVariance	wavelet.HLH_firstorder_Kurtosis	−0.232	Weak
wavelet.HLL_glcm_MCC	wavelet.HLH_firstorder_Kurtosis	0.189	Weak
wavelet.HLL_glcm_MCC	original_shape_MajorAxisLength	0.177	Weak
wavelet.HHL_glcm_InverseVariance	wavelet.HLL_glcm_MCC	−0.123	Weak
wavelet.HHL_glcm_InverseVariance	wavelet.HHH_glcm_Correlation	0.097	Negligible
wavelet.HHH_glcm_Correlation	wavelet.HLL_glcm_MCC	0.081	Negligible
